# Corneal perforation in the setting of NK/T-cell lymphoma-associated hemophagocytic lymphohistiocytosis

**DOI:** 10.1016/j.ajoc.2025.102487

**Published:** 2025-12-04

**Authors:** John R. Mark, Jeffrey H. Ma

**Affiliations:** Tschannen Eye Institute at University of California, Davis, 4860 Y St Suite 1E, Sacramento, CA, 95817, USA

**Keywords:** Hemophagocytic lymphohistiocytosis, Extranodal natural killer/T-cell lymphoma, Cornea, Corneal perforation, Corneal melt

## Abstract

**Background:**

Hemophagocytic lymphohistiocytosis (HLH) is a syndrome of severe systemic inflammation caused by pathologic immune dysregulation and resulting in multiorgan destruction^4^. While ocular manifestations are recognized in HLH, corneal perforation has not been well-described in published reports.

**Case presentation:**

We report a case of a 26-year-old male who developed a spontaneous corneal perforation in the setting of NK/T-cell lymphoma-associated HLH. The patient was transferred to our hospital with a progressively enlarging fungating facial mass and severe bilateral eyelid edema, as well as fevers, sepsis, pancytopenia and transaminitis. Biopsy of the facial mass was consistent with extranodal natural killer/T-cell lymphoma (ENKTL), and the patient was diagnosed with HLH presumed secondary to his malignancy. Ophthalmic examination revealed a corneal perforation of his left eye.

**Management and outcome:**

The patient was taken to the operating room for repair of the perforation with a corneal patch graft. Postoperative management included topical corticosteroids and antibiotics. After surgery, the patient's left eye visual acuity was 20/100. Three months later, his visual acuity was 20/30 and the corneal patch graft appeared well-healed.

**Conclusion:**

This case highlights the potential for HLH to cause corneal perforation as a severe ocular complication. Prompt diagnosis and multidisciplinary management are crucial for optimizing outcomes in such complex cases.

## Highlights

• Hemophagocytic Lymphohistiocytosis commonly affects the eyes and can be vision-threatening.
• A rare complication of hemophagocytic lymphohistiocytosis is corneal perforation.
• Corneal perforation in this population can be treated successfully with patch grafts.

## 1 Introduction

Hemophagocytic lymphohistiocytosis (HLH) is a rare, life-threatening syndrome characterized by uncontrolled activation of the immune system and presents with febrile illness, cytopenia, splenomegaly, hemophagocytosis, and other multiorgan involvement.^4^ It can develop secondary to infection, malignancy, immunodeficiency, and rheumatologic disease. Various ocular manifestations of HLH have been described, including orbital cellulitis, dacryocystitis, conjunctivitis, keratitis, retinal hemorrhages, serous retinal detachment, retinitis, uveitis and optic disc swelling.^3^^,^^5^^,^^6^ Reports of corneal perforation associated with HLH appear to be limited or absent in the published literature. This case report discusses the clinical presentation, management, and outcome of a patient with extranodal natural killer/T-cell lymphoma (ENKTL)-associated HLH who developed corneal perforation.

## 2 Case Presentation

Our patient was a 26-year-old white male with a history of angioedema and no significant ocular history, who was transferred to University of California Davis Medical Center from an outside facility with a severe, rapidly progressive, necrotizing facial infection. He reported that he had sustained a laceration to his nose one month prior to presentation. He was initially treated with oral antibiotics due to concern for infection with some improvement. However, he then developed progressively worsening facial and eyelid swelling and a rapidly enlarging necrotic facial mass with pain and was subsequently transferred to our facility. Upon arrival, the patient fulfilled diagnostic criteria for HLH, with systemic work-up demonstrating prolonged fever, splenomegaly, cytopenia (leukopenia, anemia, and thrombocytopenia), hypertriglyceridemia (446 mg/dL; normal <150), and markedly elevated ferritin (29,722 ng/mL) and soluble IL-2 receptor levels (5881 U/mL). Our ophthalmology service was consulted due to extensive swelling of his eyelids that prevented him from opening his eyes.

**Fig. 1:**
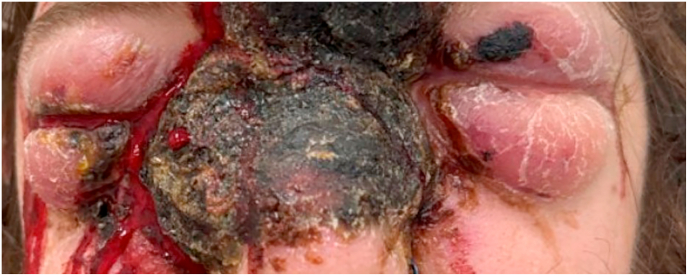
Facial mass on presentation; note the severe lid edema.

**Fig. 2:**
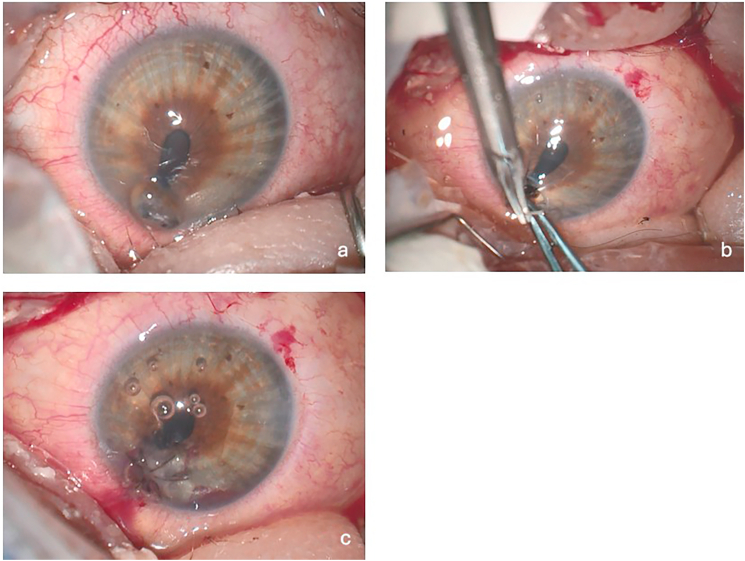
a- Inferonasal perforated cornea and iris incarceration; note the absence of corneal infiltrate b - Suturing patch graft c - Corneal patch graft secured in place with nylon suture.

Ophthalmic evaluation was challenging and limited by severe eyelid edema and pain; however, initial examination revealed a visual acuity of about 20/200 in both eyes. Intraocular pressure (IOP) was unable to be obtained in either eye due to eyelid edema despite best efforts to open the lids using Desmarres lid retractors. External exam showed an extensive necrotic midline facial mass and significant periorbital edema (Fig. 1). There was chemosis and mild conjunctival injection bilaterally. The remainder of the anterior segment exam of the right eye was unremarkable. Examination of the left cornea revealed a perforation near the inferonasal limbus with iris incarceration (Fig. 2a). Notably, there was no corneal infiltrate observed in the area of the perforation. The left anterior chamber was flat, and the pupil was peaked inferonasally towards the perforation. Dilated fundus examination (DFE) was deferred at this time. Although there was no corneal infiltrate on exam, cultures of his facial mass were positive for *Pseudomonas aeruginosa*, so fortified tobramycin antibiotic drops were initiated prophylactically.

The patient was brought to the operating room urgently for repair of his corneal perforation. Upon closer examination of the left eye under general anesthesia, a 3-mm diameter corneal perforation was noted near the inferonasal limbus with iris prolapse. There was no adjacent corneal infiltrate, and the remainder of the cornea was clear. A limbal paracentesis was created superotemporally and the anterior chamber was deepened with viscoelastic. A 3-mm hole punch was used to fashion a corneal tectonic patch graft with corneal donor tissue. The graft was then sutured into the area of perforation (Fig. 2b and c). The prolapsed iris tissue was reposited as best as possible and the viscoelastic was replaced with balanced salt solution. Subconjunctival dexamethasone and cefazolin were administered. Neomycin/Polymyxin B/Dexamethasone ointment was applied, and the eye was patched and shielded. Lastly, the fellow eye was examined with an indirect ophthalmoscope and found to be normal with no signs of corneal thinning.

**Fig. 3:**
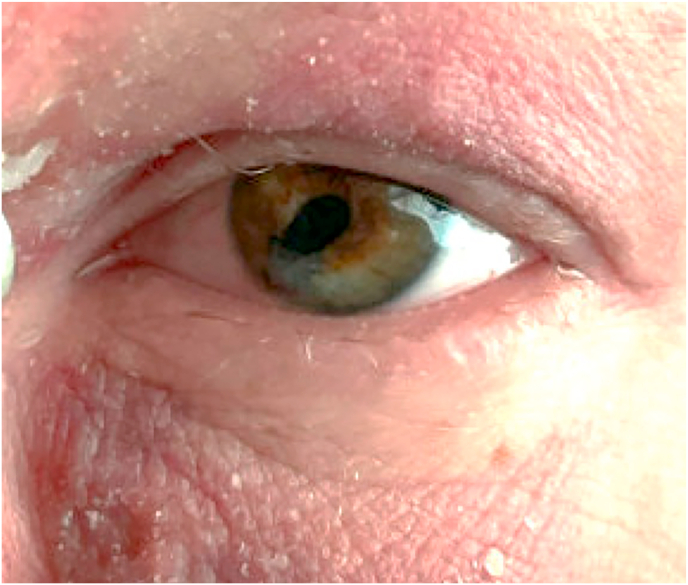
Healed corneal patch graft at postoperative month 3.

**Fig. 4:**
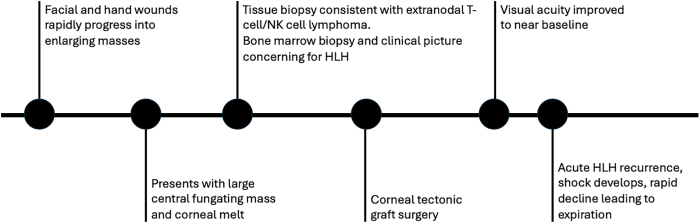
Timeline of events.

One week postoperatively, the patient's left eye visual acuity was 20/100 and the IOP was 10 mm Hg (via iCare). At three months postoperatively, visual acuity of the left eye improved to 20/30 and the corneal patch graft remained well-healed with no further signs of thinning (Fig. 3) (see Fig. 4).

## 3 Discussion and conclusions

### 3.1 Discussion

Hemophagocytic lymphohistocytosis (HLH) is a rare life-threatening systemic inflammatory condition of histiocyte and lymphocyte hyperstimulation leading to multi-organ failure. Ocular manifestations of HLH are relatively common, with a retrospective study showing that 39.0 % of patients with HLH exhibit ocular abnormalities.^8^ Our case of a sterile corneal perforation in a patient with HLH is particularly noteworthy as published accounts of corneal melt in HLH are limited or absent. The underlying mechanisms of HLH that contribute to severe ocular manifestations are not well-understood due to the paucity of histology of ocular tissues in HLH. However, existing adult autopsies have shown histiocyte infiltration of uvea, trabecular meshwork, retina, and optic nerve.^7^ In this patient, corneal perforation was unlikely caused by infection, as there was no sign of corneal infiltrate or infectious keratitis despite nearby facial soft tissue cultures growing *Pseudomonas.* There was no known preceding trauma to the eye, and the patient's extensive eyelid edema precluded development of exposure keratopathy. We propose that the corneal perforation resulted from a mechanism of histiocyte-induced destruction similarly seen in other ocular tissues in HLH.

While a histiocyte-driven process of tissue destruction is the most likely explanation for corneal perforation in this case, other mechanisms of keratolysis may also contribute. One potential pathway is an excessive increase in corneal matrix metalloproteinases (MMPs) in response to inflammation, disrupting the balance between MMPs (in particular collagenases) and their inhibitors and thereby weakening stromal architecture. Immune-complex deposition may also promote localized corneal inflammation and tissue fragility, as seen in other inflammatory disorders such as peripheral ulcerative keratitis. In addition, the hypercytokinemia seen in HLH may exert direct cytotoxic effects on keratocytes, impairing corneal integrity. Taken together, these mechanisms highlight the complex interplay of immune dysregulation in HLH.

Diagnosing and managing ocular complications in HLH is challenging due to overlapping symptoms and the severity of systemic involvement. Patients with HLH require a multidisciplinary approach to manage their multi-organ disease. In this case, the cause of the patient's facial lesions was unclear at the time of intervention, and there was significant concern that any tissue trauma could exacerbate his rapidly growing necrotizing lesions given his history of minor injuries leading to severe skin response. There was considerable concern regarding possible airway compromise during intubation with general anesthesia, as any trauma, even minor, could potentially cause a similar reaction.

Ultimately, the patient was diagnosed with extranodal natural killer/T-cell lymphoma (ENKTL) in the setting of Epstein-Barr Virus (EBV) viremia, the most common cause of acquired HLH.^4^ ENKTL is a rare aggressive lymphoma with a low incidence in the United States comprising only 0.2–0.4 % of newly diagnosed non-Hodgkin lymphomas^9^ and more commonly presents in Asian and Hispanic individuals. Although our patient was a young white man, it should be noted that racial patterns appear to bare no influence on overall survival rates.^1^

If the diagnosis of ENKTL is known, particular preoperative and intraoperative risks should be recognized. As noted previously, this patient showed a propensity to bleed and form large necrotic growths from innocuous wounds. ENKTL is angiocentric and angiodestructive, which leads to necrosis and release of cytotoxic proteins by malignant natural killer cells causing an exuberant inflammatory reaction in response to trauma.^2^ In our patient's case, the anesthesia team decided to cautiously trial endotracheal intubation with pre-emptive platelet and cryoprecipitate transfusions at the beginning of the procedure to minimize risk of bleeding. With respect to ophthalmic surgical considerations, risk for corneal graft rejection and failure may be higher than typical.

Despite these challenges, the surgical repair of the corneal perforation with a donor corneal patch graft was uncomplicated. Postoperative care included topical steroids, antibiotics, and eye shielding. A prolonged topical steroid taper was followed postoperatively given the possible increased risk of immune-mediated tissue rejection. The patient's postoperative recovery showed visual improvement from 20/200 to 20/30 at postoperative month 3. This case underscores the importance of thorough ophthalmic examinations in HLH patients to detect severe, sight-threatening complications early.

### 3.2 Conclusion

To summarize, this case presents a unique ophthalmic complication of a sterile corneal perforation in a patient with HLH. Despite routine surgical management, unique challenges emphasize the importance of a multidisciplinary approach in treating patients with this life- and sight-threatening syndrome. Early recognition of ocular complications in HLH is crucial to prevent severe morbidity. The clinical implications of this case underscore the need for healthcare professionals, especially ophthalmologists, to be aware of HLH's ocular presentations. Prompt and coordinated specialist care is essential to address potential complications like corneal perforation, which, though rare, carry significant morbidity and mortality. This case aims to enhance understanding of HLH and its potential complications, promoting timely and effective intervention to preserve vision and improve patient outcomes.

## CRediT authorship contribution statement

**John R. Mark:** Writing – review & editing, Writing – original draft. **Jeffrey H. Ma:** Writing – review & editing, Supervision.

## Patient consent

Consent to publish this case report has been obtained from the patient(s) in writing.

## Claim of priority

After conducting a literature review on 6/18/2025 utilizing PubMed and Google Scholar using the key words “(((corneal perforation) OR (corneal melt)) OR (corneal rupture)) AND (hemophagocytic lymphohistiocytosis)”, we did not find any prior reports of corneal perforation in the setting of HLH.

## Authorship

All authors attest that they meet the current ICMJE criteria for Authorship.

## Declaration of generative AI and AI-assisted technologies in the writing process

During the preparation of this work the author(s) used ChatGPT to improve readability. After using this tool/service, the author(s) reviewed and edited the content as needed and take(s) full responsibility for the content of the published article.

## Funding

No funding or grant support.

## Declaration of competing interest

The authors declare that they have no known competing financial interests or personal relationships that could have appeared to influence the work reported in this paper.
